# When a Whole Practice Model Is the Intervention: Developing Fidelity Evaluation Components Using Program Theory-Driven Science for an Integrative Medicine Primary Care Clinic

**DOI:** 10.1155/2013/652047

**Published:** 2013-11-27

**Authors:** Sally E. Dodds, Patricia M. Herman, Lee Sechrest, Ivo Abraham, Melanie D. Logue, Amy L. Grizzle, Rick A. Rehfeld, Terry J. Urbine, Randy Horwitz, Robert L. Crocker, Victoria H. Maizes

**Affiliations:** ^1^Arizona Center for Integrative Medicine, College of Medicine, University of Arizona, P.O. Box 245153, Tucson, AZ 85724-5153, USA; ^2^Health Unit, The RAND Corporation, Santa Monica, CA 90407-2138, USA; ^3^Center for Health Outcomes & PharmacoEconomic Research (HOPE), College of Pharmacy, University of Arizona, P.O. Box 245153, Tucson, AZ 85724-5153, USA; ^4^College of Nursing, University of Arizona, P.O. Box 245153, Tucson, AZ 85724-5153, USA

## Abstract

Integrative medicine (IM) is a clinical paradigm of whole person healthcare that combines appropriate conventional and complementary medicine (CM) treatments. Studies of integrative healthcare systems and theory-driven evaluations of IM practice models need to be undertaken. Two health services research methods can strengthen the validity of IM healthcare studies, practice theory, and fidelity evaluation. The University of Arizona Integrative Health Center (UAIHC) is a membership-supported integrative primary care clinic in Phoenix, AZ. A comparative effectiveness evaluation is being conducted to assess its clinical and cost outcomes. A process evaluation of the clinic's practice theory components assesses model fidelity for four purposes: (1) as a measure of intervention integrity to determine whether the practice model was delivered as intended; (2) to describe an integrative primary care clinic model as it is being developed and refined; (3) as potential covariates in the outcomes analyses, to assist in interpretation of findings, and for external validity and replication; and (4) to provide feedback for needed corrections and improvements of clinic operations over time. This paper provides a rationale for the use of practice theory and fidelity evaluation in studies of integrative practices and describes the approach and protocol used in fidelity evaluation of the UAIHC.

## 1. Introduction

Integrative medicine (IM) may be defined as a clinical paradigm that is patient-centered, healing-oriented, and embracing of appropriate therapeutic approaches whether they originate in conventional or complementary medicine (CM). IM reaffirms the importance of the therapeutic relationship, focuses on the whole person and lifestyle, and renews attention to healing [1]. IM services are typically delivered by an interprofessional team of conventional and CM practitioners who provide a comprehensive and seamless continuum of whole person care [2]. However, there is great variability in the organization of integrative medical practices and delivery of IM services [3–8]. While studies suggest clinical- and cost-effectiveness of some practice models [3, 9, 10], a major drawback has been a primary focus on measuring the outcomes of the treatments and a lack of focus on documenting and measuring the processes and mechanisms of the whole IM practice model itself that generated those outcomes.

The Institute of Medicine Summit on Integrative Medicine and the Health of the Public called for IM to move beyond evaluation of CM therapies toward studies of integrative healthcare systems [11]. To do so, theory-driven evaluations of IM practice models capturing the mechanisms of the overall practice need to be undertaken. Typically, descriptions of IM practices have used nonspecific language such as “a practice that provides IM care” or have offered a list of features valued by IM such as patient-centered care, increased visit time, and use of conventional and CM therapies [6]. Such descriptions do not sufficiently identify the evaluable components and processes that are organized within a practice model to a level where measurable definitions can be employed. To be methodologically valid and replicable, studies ideally should attend to the internal assumptions, components, and processes of the practice itself and how these may (or may not) contribute to health and/or cost outcomes. Factors that may account for this weakness in IM studies include challenges in the design and execution of complex program evaluations as well as lack of uniform definitions, operational criteria, and quality standards for IM overall. Two health services research methods can strengthen the validity of IM healthcare studies, practice theory, and fidelity evaluation.

*Practice Theory*. Complex interventions require theoretical understanding of how they cause change so that weaknesses can be identified and improved [12]. A practice theory, also known as a program theory or logic model, is a representation of how a complex intervention is supposed to work [13, 14]. A conceptual map, a practice theory is formal, specific to the practice under study, and includes definitions and explanations about how the mechanisms of the practice as a whole work internally. Thus, practice theories specify and define the interrelated components of a practice—those underlying assumptions, values and goals, structures, and processes that are expected to lead to the desired patient outcomes [15, 16].

Practice theories have seldom been made explicit in IM research. Studies tend to focus on outcomes of CM treatments or on qualitative impressions. Consequently, most studies of IM models of care are “black-box evaluations” providing little insight into what caused the outcomes or why; that is, what is inside the box. Even many well-known lifestyle change programs do not examine the impact of individual program components [17]. With no practice theory, there is no conceptual framework to allow assessment of the relative influences of the independent variables on the outcomes. In turn, this contributes to problems of internal validity, generalizability, and, consequently, replicability (model validity) [18].

Model validity is not unique to IM yet is especially important. Simply adding CM to conventional care is not IM. Rather, IM is a larger paradigm for a process of patient-centered, whole person healthcare [19]. Model validity is also important when incorporating CM modalities from healing systems other than the biomedical paradigm. These require particular consideration as to their fit among the clinical and healing philosophy(ies) involved, the structures and processes of patient care, the business and financing dimensions of the practice, and the expected outcomes on the patient and costs of care [20, 21].

*Fidelity Evaluation*. Once theoretical components of a practice are specified they can be logically evaluated. Fidelity evaluation is a practice-level method that serves purposes similar to treatment adherence in clinical studies. Fidelity measurement assesses integrity of initial and ongoing implementation (i.e., it is the practice model being delivered according to design), yields potential covariates between the model and its outcomes, assists in the interpretation of findings; supports external validity and replication, and serves as quality improvement feedback [22].

How faithfully an intervention is implemented as it was planned affects how well it succeeds and how reliably it can be adapted to other settings [23]. The risk of attempting an intervention study without assessing fidelity is that it becomes impossible to determine whether modest or negative findings are due to poor implementation (a type III error—wrongly concluding an intervention had no impact when it was actually not implemented or not implemented as planned) or to weaknesses in the model [24] that may be improved.

Fidelity is assessed by a process evaluation tailored to the practice theory and can involve multiple data sources including questionnaires, administrative records, and qualitative observations. Five dimensions of fidelity evaluation provide a comprehensive picture of program integrity. *Differentiation* is the assessment of essential components against the outcomes to discover those components that made a difference [22]. This is particularly important for IM, a field rife with components thought to be essential, but which are difficult to assess (e.g., supporting the body's innate healing capacity). *Adherence*, the bottom-line measurement of fidelity, refers to whether all components are being delivered as designed. *Dose* is the amount of an intervention received by patients compared to what was intended and is typically measured by frequency and duration of services utilized. Dose is an important dimension for many treatment interventions (e.g., a course of acupuncture) but is difficult to define for individualized multimodality interventions such as IM. *Quality of delivery *addresses the concern that if a model was delivered inadequately, the degree of fidelity achieved would be adversely affected. *Participant responsiveness*, also to be considered, is the extent to which patients participate in and/or find the services offered by the practice to be helpful [22, 25].

The purpose of this paper is to describe the approach used in defining a practice theory for use as the basis for identifying the concepts to measure in fidelity evaluation of a new IM primary care clinic. It is hoped that this example can help advance the methodological rigor of integrative healthcare research study design.

### 1.1. Intervention

The Integrative Medicine Primary Care Trial (IMPACT) is an evaluation of an IM primary care clinic in Phoenix, AZ, the University of Arizona Integrative Health Center (UAIHC). A community-based private practice operated in affiliation with District Medical Group, UAIHC provides adult primary care fully integrated with CM services delivered by a physician-led interprofessional team. The clinical approach combines disease management, prevention, and health promotion by attending to the prevention or treatment of acute and chronic illnesses while emphasizing reduction of modifiable risk factors. Practitioners include IM-trained family physicians and a physician assistant, nurses, an acupuncturist (traditional Chinese medicine), a chiropractor (manual medicine), a behavioral health clinician (mind-body medicine), a nutritionist, and a health coach. UAIHC is financed by a hybrid revenue structure combining health insurance reimbursement with membership fees paid by patients and/or employer contributions.

The UAIHC model was defined over a five-month period by a committee convened by the Arizona Center for Integrative Medicine (AzCIM). The committee included IM physicians (three family physicians, an internist, and a surgeon, several with training in CM modalities), a diplomat of oriental medicine, the AzCIM business manager, and a development officer. The committee chose to develop a primary care clinic, rather than a specialty clinic as is frequently done in integrative medicine. This decision was based on an interest in addressing the lifestyle medicine needs (not typically addressed in conventional primary care clinics) of patients with costly chronic conditions. The committee reviewed a business plan, developed by a physician consultant previously trained through the AzCIM Fellowship in IM and the business manager. Focus groups with consumers, physicians, and donors were conducted and their input incorporated into the plan.

IMPACT was approved by the University of Arizona Institutional Review Board and is registered in clinicaltrials.gov (no.NCT01785485). The full protocol is found elsewhere [26].

## 2. Materials and Methods 

The methods used to develop the practice theory for fidelity evaluation of this study included identifying components of the UAIHC model, examining the relevant literature, and collaborating with experts and planners to define how each would be measured.

A process evaluation protocol was then developed to assess fidelity for four purposes: (1) as a measure of intervention integrity, that is, to determine whether the intervention/practice was delivered as intended; (2) because integrative primary care has not previously been defined and evaluated, and since the clinic under study is developing and continuously being refined, fidelity data will also be used descriptively to follow its progress; (3) to examine components of the model as potential covariates in the outcomes analyses, assist in interpretation of findings, and for external validity and replication; and (4) to provide feedback to model developers and clinic personnel as to how the clinic model is being implemented by staff and perceived by patients to allow for corrections, improvements, and fine tuning of clinic operations over time.

The fidelity sample is drawn from two groups, from patients (*n* = 180) enrolled as members of UAIHC and from clinic personnel (*n* = 15–20). Data are collected 10 times across two years of the study (monthly for the first six months, then quarterly for six months, and then semiannually). Data are collected through a self-administered questionnaire to all patients seen at the clinic on a single randomly chosen day of each data collection period to assess patient experiences of the UAIHC model. A self-administered practitioner experiences questionnaire is conducted with all clinic personnel, and random audits of deidentified patient medical records and billing data both also occur during the same time periods.

### 2.1. Identifying Practice Theory Components

The first step of theory-driven evaluation is to make the theory explicit. Often, this is done using methods such as concept-mapping or other social science techniques [14]. Absent these, source documents (e.g., strategic plans, legislated requirements) are viable alternatives [27]. The source document used for IMPACT was the UAIHC business plan. While the business plan presented the clinic's approach to areas such as vision, goals, marketing, finance, and human resources; through careful translation, it provided quantifiable definitions for the practice theory. Model components were also identified through collaboration and dialogue with the planning committee and model developers.  Table 1 shows the UAIHC components as stated in the business plan and how these were refined into measurable constructs for evaluation.

### 2.2. Identifying Measures of Each Component for Fidelity Evaluation

The business plan stated that UAIHC adheres to the philosophies and principles of IM and evidence-based primary care. From the literature, a definition of IM (referenced in the plan) was used to identify philosophic concepts [28]; IM principles used were those identified by AzCIM (http://integrativemedicine.arizona.edu/about/definition.html) and adapted for the IOM Summit on IM [1]. Philosophies and principles of general primary medical care were derived from reports by the IOM [28, 29]. Literature on the patient centered medical home (PCMH), a framework for excellence in primary care [30, 31], was also used (Table 2).

Several IM philosophies and principles overlapped those of primary care and the PCMH, and several were conceptually distinct. Additionally, some components required expansion and reorganization. The final components were reviewed with two members of the clinic planning committee, the AzCIM medical director and the business plan developer (Table 1). Once definitions were determined, measures were identified. Where possible, measures with established psychometric properties and used in other primary care or IM studies were chosen. For some components, particularly those unique to IM, items or indicators were developed for the study.  Table 3 aligns the components and measures.

### 2.3. Practice Theory Components

#### 2.3.1. Integrated Care (Comprehensive and Coordinated) Including CM Interventions

In primary care, integration is an omnibus term for comprehensive and coordinated services that provide seamless and continuous care. Comprehensive refers to services directly provided or arranged by a physician/other practitioner for any health problem at any life stage including ongoing care of patients in various settings (e.g., hospitals, nursing homes, and clinicians' offices) [29]. Coordinated refers to linkages among practitioners and services resulting from a coherent treatment plan within a single episode of care and over a longer period and across systems and settings [29]. In IM, integration of evidence-based CM modalities is a cardinal feature; one IM study for low back pain found that patients receiving coordinated IM team care had greater improvements in functioning and pain scores than controls [37].

For IMPACT, integrated care is defined as comprehensive and coordinated services inclusive of CM modalities. It is assessed in several ways: a single item measures patient ratings of coordination (i.e., “how well is your care coordinated among all practitioners involved…? Coordination means that all UAIHC practitioners you see are familiar with your treatment plan”); medical records data assess the number of referrals by type both within and outside UAIHC (comprehensiveness) and the type and frequency of conventional and CM modalities recommended/prescribed in the treatment plan (integration). Billing data also assess the type and frequency of conventional services billed to insurance and CM services used from UAIHC membership plans.

#### 2.3.2. Prevention and Health Promotion Services Together with Treatment and Disease Management

Like comprehensive primary care [29], IM includes prevention and health promotion as well as diagnosis, treatment, and management of clinical conditions [38]. For primary prevention, services may include dietary counseling, mind-body techniques, or interventions to improve sleep quality. For secondary prevention, stress management and nutritional recommendations (e.g., teaching relaxation practices or prescribing a Mediterranean diet to reduce risk of heart attack) may be used, as are interventions that attenuate risks of conventional therapies (e.g., coenzyme Q10 to mitigate statin-related myalgia) [39]. In tertiary prevention, these same interventions relate to such goals as pain management, symptom control, stress relief, and reduced disease progression [38]. One study of an intensive IM intervention combining dietary and lifestyle counseling with nutritional supplements showed significant reductions in 10-year cardiovascular risk and frequency of metabolic syndrome as compared to controls [40].

Five items from the Ambulatory Care Experience Survey (ACES) assess patient experiences of prevention and health promotion [33]. Medical records data assess the number of health promotion/prevention services recommended by practitioners. Billing data assess the number of health promotion/prevention classes and groups attended through UAIHC membership plans.

#### 2.3.3. Natural and Less Invasive Treatments and Interventions

Use of interventions that are natural and minimally invasive is an IM principle [1] and includes such modalities as diet, botanical medicine, manual medicine, and mind-body approaches. Recommendations of natural products and/or lifestyle interventions before or concurrent with prescriptions and invasive procedures are assessed by counts obtained from the medical record.

#### 2.3.4. Whole Person Care

Despite widespread use of the term “whole person care,” medicine has no clear model of a whole person [41]. IM defines a whole person as an indivisible organism, in an environmental context, with patterns of dysfunction within the entire person rather than just localized symptoms [42]. Such interconnectivity requires that all factors influencing health, wellness, and disease be considered, including body, mind, spirit, and community [1]. Whole person is also embraced by primary care although somewhat differently. The primary care definition of whole person is care that addresses the majority of personal health care needs—all problems, unrestricted by problem or organ system, and including physical, mental, emotional, and social concerns [29].

Patient experiences are assessed by three items about whole person care, one each from the ACES [33], the Consumer Assessment of Healthcare Providers and Systems (CAHPS) [34], and the Consultation and Relational Empathy (CARE) [35] instruments. Practitioner experience of providing whole person care is assessed by one item about developing whole person integrative treatment plans. It is also assessed by evidence of a whole person review of systems in the medical record (e.g., of family/social life, stress, mental health, health habits, diet, physical activity, and spirituality/religion).

#### 2.3.5. Healing Orientation to Support the Individual's Innate Healing Capacity

Healing, an IM principle, refers to a patient's recovery, repair, and restoration; it does not necessarily include a cure [35]. It relates to another IM principle, that of support for the body's innate healing capacity, the inherent self-organizing, homeostatic, and healing ability of living systems to establish, maintain, and restore health. In IM, a central role of a practitioner is to facilitate and augment this process, identify and remove obstacles to health and recovery, and support a healthy internal and external environment [43, 44] (e.g., discussion with the patient of the body's healing abilities or use of probiotics concurrent with antibiotic treatment to support a healthier microbiome) [45]. Medical records data are assessed for instances of treatments to support the body's healing capacity.

#### 2.3.6. IM Practitioners Exemplify the Principles and Commit to Self-Exploration and Self-Development

This IM principle holds that it is difficult to facilitate health and healing in others if practitioners have not explored this for themselves, and that self-reflection resulting in health for the clinician should be encouraged. Four items developed for the study assess practitioner experiences of self-care being valued and supported by the team and whether time has been taken to engage in self-care activities.

#### 2.3.7. Patient-Centered Partnership

Patient-centered care is a standard for high-quality interpersonal care [46]. Five areas of physician communication behavior are generally assessed: (1) understanding the patient's illness within the biopsychosocial (whole person) context; (2) eliciting understanding and validating the patient's reasons for the visit, illness perspective, and information needs; (3) helping the patient understand the problem and its treatment; and (4) creating a partnership (alliance) wherein patients share in decision making and responsibility. As a result, patients may experience increased trust in their providers and greater health self-efficacy [47, 48].

In IM, a patient-practitioner partnership with clear responsibilities supports shared decision-making leading to customized treatment recommendations in response to patients' preferences. One study of a personalized IM mind-body treatment plan for cardiovascular risk developed through shared decision-making between the patient and practitioner showed significant reduction in 10-year coronary heart disease risk as compared to usual care [49]. UAIHC facilitates this relationship through use of a Health Partnership Acknowledgment (HPA) signed by both patient and physician. In the HPA, both commit to upholding their roles to achieve adequate physical activity; healthy diet, sleep, body weight, and relationships; avoiding harmful habits; managing stress; and maintaining life balance. Extended visit length (90 minute initial appointments with 30 minute followups) is also seen as an element of patient-centered IM care [1].

For the practice theory, patient-centeredness encompasses practitioner communication style (listening, understanding, explaining, validating, showing empathy, and sharing decision-making and treatment planning), a patient-practitioner partnership (signed HPA), adequate visit time, and patient trust. Patient experiences of practitioner communication, adequacy of time spent, and trust are assessed by items from the CAHPS [34], and practitioner empathy is assessed by the CARE [35]. Evidence of a signed HPA and length of visit are obtained from medical records.

#### 2.3.8. Provision of Other Services Not Typically Provided in Primary Care

UAIHC offers a variety of educational classes and groups (e.g., diet education, meditation, and yoga) for inclusion in a patient treatment plan or as chosen by the patient. Group visits for primary care are reimbursed through the patient's health insurance. Medical records data assess the number of UAIHC educational classes/groups and group visits recommended in the treatment plan. Billing data assess the number of educational classes/groups utilized through the membership plans and group visits billed to insurance.

#### 2.3.9. Integrative Team Care with Health Coaches

Like the PCMH [30], UAIHC utilizes an interdisciplinary physician-led team. However, combining conventional medicine clinicians with CM practitioners is what differentiates IM from other models of team-oriented healthcare. Integrative teams are far more complex than other teams because they are faced with differences in disciplines as well as philosophies of health and healing. An integrative team is characterized by a cohesive blend of practice philosophies with patient services through consensus building, mutual respect, team conferences, and a shared vision of healthcare permitting each member to contribute their knowledge and skills within a shared treatment plan [50].

Team cohesiveness (vision sharing, safety in participation, shared concern for excellence, and support for innovation) is assessed by the short Team Climate Inventory (TCI) [36]. Five additional items measure practitioner experiences of an IM team—shared treatment planning, patient preferences, whole person care, treatments supporting body's healing capacity, and equal consideration all team members' recommendations. A single item screener taps occupational stress and burnout [51]. An open-ended item asks practitioners to reflect on how the UAIHC team experience has affected the clinical care they provide. Evidence of an identified patient team and documentation of complex patients being the subject of team meetings is obtained from the medical record and meeting documents.

#### 2.3.10. Hybrid Financing

Innovative healthcare models are being created in which patients pay membership fees for expanded treatment options and enhanced services not covered by insurance. Studies of these financing structures find that patients report enhanced staff interactions, greater access to care, and better care coordination [52].

UAIHC's hybrid financing model combines insurance reimbursements with a membership fee. The membership covers visits with CM practitioners, health coach, and educational classes/groups. Fees are based on three annual cost tiers: (1) basic (5 visits with the patient's choice of CM practitioners, 2 visits with a health coach, and 2 classes/groups); (2) core (10 visits with CM practitioners, 4 health coach visits, and 4 classes/groups); and (3) expanded (20 CM visits, 8 health coach visits, and 8 classes/groups). Patients may change tiers and may purchase additional CM or health coach visits at a discount. Assessment is from administrative and billing data documenting the membership tier chosen, frequency of tier changes, purchase of additional visits, rate of member drop out, and utilization rate of tier benefits.

#### 2.3.11. Enhanced Access to Healthcare

Access in primary care is the ease with which a patient can initiate a clinical interaction for any health problem and includes reduction of administrative and financial barriers such as through shorter wait times for appointments and same day appointments when warranted [29]. In the PCMH, access is similarly defined as care within an appropriate timeframe [30]. Patient experiences of enhanced access are assessed by an item about time between the patient's initiation of service and receipt of an appointment (most recent visit) and two items from the ACES [40] about courtesy and helpfulness of front desk staff.

*Analysis of Fidelity Data*. The data gathered during the fidelity evaluation will be used to generate descriptive statistics for each practice theory component for each time period and over time. As discussed above, these results will be made available on a regular basis to clinic staff as feedback on clinic operations and will be used in the analysis and interpretation of clinic outcomes.

## 3. Results and Discussion

For this study, the complexity of the intervention (IM), and the fact that integrative healthcare has few uniform definitions, operational criteria, and quality standards to guide evaluation, necessitated that the fidelity of the intervention itself be monitored in addition to its outcomes. Whereas primary care and the PCMH have well-defined processes of healthcare delivery, IM is a young field. There are very few integrative healthcare effectiveness studies and fewer still that consider practice theory and fidelity evaluation.

In our approach we considered using a conceptual framework such as the well-established Donabedian structure, process, and outcome framework often used in quality of care assessments [53, 54]. However, this framework does not include a dimension for philosophy. In integrative medicine, the underling philosophical rationale is cardinal and is derived from diverse medical systems both conventional and complementary. These philosophical components of IM are difficult to institute and to measure. While we defined and incorporated such philosophies in our fidelity evaluation measures, these measures may need to be modified as the clinic continues to develop and as IMPACT data are analyzed and interpreted. Further, although we are directly measuring adherence, and will be able to assess differentiation during our data analyses, the other dimensions of fidelity such as dose, delivery quality, and participant responsiveness may only be partially testable until, and if, decisions are made as to the optimal levels of these in our IM model.

Another way to look at the challenge in assessing novel models of clinical care revolves around the codevelopment of the clinical model and the research protocol. Most often, a clinical model is developed, shown to meet defined standards, and then rigorously assessed. For IMPACT, researchers and UAIHC developers collaborated regularly from the outset of the planning stage. The UAIHC model was designed to be true to its business plan and to definitions of the field incorporated within it. However, the only source document available for developing the practice theory was the business plan. Not written for research purpose, generating measureable constructs encrypted within the business plan required significant effort. As an object lesson, it is important that practice model designers remain aware of evaluation needs, and that collaboration begins early.

Together, these challenges to complex clinical research design force a careful evaluation of outcome results. Even the best research design will only partially test a clinical model, and this study is no exception. However, as findings become available, refinements can be made. Future IM studies that use a similarly developed practice theory may eventually lead to a fully testable framework, one critically needed by IM.

## 4. Conclusion

Integrative medicine is poised to make significant contributions to U.S. healthcare. However, studies that measure only the outcomes of treatments, and not the processes of their delivery, risk devaluing the tenets of IM and homogenizing its unique identity into the paradigm of symptom management currently dominating American healthcare. Studies of IM models of care that develop practice theories and monitor fidelity may provide the evidence needed to expand uptake of IM into the U.S. healthcare system.

## Figures and Tables

**Table 1:** Initial business plan components and final practice theory components.

Initial business plan components	Practice theory components
Philosophies and principles of IM and evidence-based primary care.	Integrated care (comprehensive and coordinated) inclusive of complementary medicine (CM) interventions.
	Prevention and health promotion services together with treatment and disease management.
	Use of less invasive and natural treatments and interventions.
	Whole person care.
	Healing orientation to support the individual's innate healing capacity.
	Practitioners exemplify IM principles and commit to self-exploration and self-development.
Patient-centered care experienced by patients as a true health partnership. Use of a Health Partnership Acknowledgement agreement.	Patient-centered partnership (practitioner communication style, mutual decision making, empathy, patient trust, adequate visit time, and Health Partnership Acknowledgement form).
Services not typically provided in primary care (educational classes/groups, group visits).	Services not typically provided in primary care (educational classes and groups, group visits).
A team care model with health coaches.	Integrative team care model with health coaches.
Hybrid financing model.	Hybrid financing model.
Enhanced access to care.	Enhanced access to care (shorter wait time to appointments, front desk helpfulness).

**Table 2:** Philosophies and principles of integrative medicine (IM) and evidence-based primary care.

Integrative medicine	
A healing-oriented medicine that considers the whole person (body, mind, and spirit), including all aspects of lifestyle. It reemphasizes the relationship between patient and physician and integrates the best of complementary and alternative medicine (CAM) with the best of conventional medicine [32]. The principles (of IM) include patient-centered care that is comprehensive, combines conventional and CAM interventions, supports the innate healing capacity of the individual, is least invasive and natural, promotes prevention as well as disease management, and is provided by an integrative health care team through a provider-patient partnership [1].	

Primary care	

Primary healthcare is the provision of healthcare services that are accessible and integrated (comprehensive, coordinated, and continuous) from clinicians who are accountable for addressing a large majority of personal healthcare needs through a sustained partnership with patients while practicing in the context of family and community [28, 29]. A patient-centered medical home (PCMH) is a team-based healthcare delivery model that provides comprehensive and continuous medicalcare to patients with the goal of obtaining maximized health outcomes. A PCMH has the attributes of first contact and continuous access to a personal physician, coordination of care, whole person orientation, a physician-led team of practitioners, quality and safety, enhanced access, and adherence to principles of patient centeredness [30].	

**Table 3:** UAIHC practice theory components and fidelity measures.

Component, subcomponent	Patient experiences questionnaire	Practitioner experiences questionnaire	Medical records audit	Administrative/billing data
Integrated care (comprehensive and coordinated) inclusive of CAM interventions				
Comprehensive			Number and type of services/referrals^a^	
Coordinated	Rating of treatment plan coordination^a^			
Integrated conventional and CAM			Type and frequency conventional and CAM prescribed^a^	Type and frequency conventional and CAM billed/used from tier^a^

Prevention and health promotion services together with treatment and disease management				
	Practitioner… Talk about prevention; help with change; ask if health interferes; help with weight and emotions^a^		Type and frequency of prevention, lifestyle interventions prescribed^a^	Number and type of lifestyle/health promotion/prevention classes and groups used from membership tier^a^

Use of less invasive and natural treatments and interventions				
			Use of natural products, lifestyle interventions before or with Rx or invasive procedures^a^	

Whole person care				
	Practitioner knows your… Medical history; responsibilities; health values and beliefs^b^; worries and stress^c^		Whole person review of systems^a^	

Healing orientation to support body's innate healing capacity				
			Use of treatments, products, to support healing capacity; treatment plans support innate healing^a^	

IM practitioners exemplify principles and commit to self-exploration and development				
		Team values self-care; healthy activity is encouraged; enough time taken for self-care; occupational stress; reflection on changes in approach to care^a^		

Patient-centered partnership				
Practitioner communication	Practitioner… Explain understandably; listen carefully; give information; know your medical history; show respect for you^c^			
Shared decision making	Practitioner discuss… Reasons for treatment; reasons against treatment; your preferences^c^			
Empathy	10-item measure of provider empathy^d^			
Trust	Can tell anything; trust with your care; tells the truth; cares about your health; cares about you; rate trust 1–10^c^			
Time	Enough time spent^c^			
Partnership			Discussion of UAIHC Health Partnership Acknowledgement (HPA) noted in chart^a^	Signed HPA in UAIHC membership file^a^

Provision of other services not typically provided in primary care				
			Number and type of UAIHC classes and groups recommended in treatment plans^a^	Number and type of classes and groups attended through UAIHC member tier^a^

Integrative team care with health coaches				
Shared vision, safety, task orient, support innovation		14-item measure of team climate^e^		
Shared philosophies of IM health and healing		Team members… Understand others' philosophies; learn different modalities together; no one left out^a^		
Integrative treatment planning		Team/treatment plan… Team collaborates well; patient priorities considered first; whole person plans; plans support innate healing; equal consideration of all team members^a^	Patient team identified in chart^a^	Complex patients documented in team meeting records^a^
Health coaches				Number of visits with health coaches used through member tier^a^

Hybrid financing model				
				Member tier chosen, frequency of tier changes, frequency of additional visits purchased, member drop out; utilization rates of tier benefits^a^

Enhanced access to care				
Timeliness of visit	Wait time to appointment^a^			
Courtesy of staff	Helpful, courteous clerks^b^			
Longer visit time	Practitioner spend enough time^c^		Duration of most recent visit^a^	Duration of most recent visit^a^

^a^Study item/variable.

^b^Ambulatory Care Experiences Survey (ACES) [33].

^c^Consumer Assessment of Health Plans Study (CAHPS) [34].

^d^Consultation and Relational Empathy measure (CARE) [35].

^e^Team Climate Inventory (TCI) [36].
